# Functionalized Biochar from the Amazonian Residual Biomass Murici Seed: An Effective and Low-Cost Basic Heterogeneous Catalyst for Biodiesel Synthesis

**DOI:** 10.3390/molecules28247980

**Published:** 2023-12-07

**Authors:** Thaissa Saraiva Ribeiro, Matheus Arrais Gonçalves, Geraldo Narciso da Rocha Filho, Leyvison Rafael Vieira da Conceição

**Affiliations:** Laboratory of Catalysis and Oleochemical, Institute of Exact and Natural Sciences, Federal University of Pará, Belém 66075-110, PA, Brazil; saraivathaissa@gmail.com (T.S.R.); matheusarrais38@gmail.com (M.A.G.); narciso@ufpa.br (G.N.d.R.F.)

**Keywords:** murici seed, biochar, biodiesel, basic heterogeneous catalyst, agroindustrial residue

## Abstract

This study presents the synthesis of a basic heterogeneous catalyst based on sodium functionalized biochar. The murici biochar (BCAM) support used in the process was obtained through the pyrolysis of the murici seed (*Byrsonimia crassifolia*), followed by impregnation of the active phase in amounts that made it possible to obtain concentrations of 6, 9, 12, 15 and 18% of sodium in the final composition of the catalyst. The best-performing 15Na/BCAM catalyst was characterized by Elemental Composition (CHNS), Thermogravimetric Analysis (TG/DTG), X-ray diffraction (XRD), Fourier Transform Infrared Spectroscopy (FT-IR), Scanning Electron Microscopy (SEM), and Energy Dispersion X-ray Spectroscopy (EDS). The catalyst 15Na/BCAM was applied under optimal reaction conditions: temperature of 75 °C, reaction time of 1.5 h, catalyst concentration of 5% (*w*/*w*) and MeOH:oil molar ratio of 20:1, resulting in a biodiesel with ester content of 97.20% ± 0.31 in the first reaction cycle, and maintenance of catalytic activity for five reaction cycles with ester content above 65%. Furthermore, the study demonstrated an effective catalyst regeneration process, with the synthesized biodiesels maintaining ester content above 75% for another five reaction cycles. Thus, the data indicate a promising alternative to low-cost residual raw materials for the synthesis of basic heterogeneous catalysts.

## 1. Introduction

In recent decades, emissions of gases that contribute to the greenhouse effect have increased due to the growing demand for energy [1]. The development of a clean, ecological, sustainable alternative that meets the needs of the market has been widely discussed by the scientific community around the world [2,3]. In this scenario, biodiesel has attracted attention because of its properties, which are similar to fossil diesel [4,5], as well as by having advantages, such as the emission of less toxic suspended particles, lower emission of smoke and byproducts of its combustion, and greater safety and production capacity from renewable sources [6].

Chemically, biodiesel is a mixture of alkyl esters of fatty acids derived from renewable sources such as vegetable oils, waste oils, animal fats, and greases [7]. This biofuel can be obtained via esterification of fatty acids or via transesterification of triglycerides with a short-chain alcohol, usually using acidic, basic, or enzymatic catalysts [8,9,10]. The application of heterogeneous catalysts in the transesterification of oils offers advantages for reducing costs in the production of biofuels because they are easy to separate, noncorrosive, reusable, and can be regenerated [11,12,13,14]. In this scenario, several supports were investigated. These supports included ZrO_2_ [15], SrFe_2_O_4_ [16], CuFe_2_O_4_ [17], TiO_2_ [18], Na_2_Ti_3_O_7_ [19], and SiO_2_ [20]. The objective was to increase the dispersion of active phases on such supports, as well as increase the reuse capacity of these catalysts. In addition, activated carbon from residual biomass can be used as a potential support [21].

Biochar is a carbonaceous material that can be obtained from the thermochemical degradation of biomass. It can be obtained through several techniques, such as pyrolysis, carbonization, liquefaction, and hydrothermal carbonization and roasting, in which the materials generated will have physicochemical characteristics from the biomass used, as well as the functionalization and activation process [22,23]. Biochar can exhibit indispensable catalytic properties, such as having customizable porous structures, excellent stability in acidic or basic media, and an extensive specific surface area [24]. Several studies of preparation of carbon-based catalysts or biochar from different residual biomasses, functionalized with several active phases, have been reported [25], such as: murumuru kernel shell [26,27,28] avocado seed [29], date seed [21], rice husk [30], pomelo peel [31], banana peel [32,33], chicken manure [34], citrus fruit peel [35], coconut coir husk [36] and coffee husk [37].

Murici (*Byrsonimia crassifolia*) is a small-sized tree native to South America and widespread throughout the Amazon region. The fruit is of the trilocular drupe type, rounded, about 1.5–2.0 cm in diameter, with the pulp constituting 70.9% of the fruit. It possesses a yellowish coloration when ripe with characteristic aroma and taste. The seed is rounded, rigid, and constitutes 29.1% of the fruit [38,39]. Fruiting occurs usually between November and May, with productivity of around 12.0 kg per tree. The murici fruit is appreciated by local populations, usually raw, and its pulp is used in the manufacturing of juices, sweets, creams, jellies, ice cream, and liqueurs [40].

Bitonto et al. [29] prepared a biochar from the pyrolysis of avocado seeds in a tubular oven at 900 °C for 2 h in an atmosphere of N_2_ and then investigated the catalytic activity with different concentrations of CaO (5, 10, and 20%). The authors concluded that the best catalytic activity in the biodiesel synthesis process was the catalyst synthesized with 20% CaO concentration. This catalyst was used in the transesterification of sunflower oil with methanol in the optimal reaction condition of temperature of 99.5 °C, reaction time of 5 h, catalyst concentration of 7.3% *w*/*w* and MeOH:oil molar ratio of 15.6:1, provided a biodiesel with ester content of 99.5%, and three reaction cycles of the catalyst heat treated in a tubular oven at 550 °C for 3 h under an atmosphere of N_2_. Based on the results obtained, the authors concluded that the increase in the concentration of calcium oxide in the biochar positively affected the basicity of the material, which consequently led to an increase in the catalytic activity in the transesterification reaction of triglycerides.

Jamil et al. [21] studied four heterogeneous carbon-based catalysts impregnated with 15% by mass of alkaline metal oxides (CaO, MgO, BaO and SrO). The carbon-based material was obtained from the tubular furnace carbonization of the dates’ residue (400 °C/5 h) produced after the extraction of their oil. The biodiesel synthesis was conducted with methanol and using four types of oils, date, coconut, palm, and waste cooking oil, at temperatures of 55–75 °C, reaction times of 0.5–2.5 h, catalyst concentration of 1–5% and MeOH:oil molar ratio of 6:1–18:1. The biodiesel yield obtained was 94.27% under the best reaction conditions and maintenance of high catalytic activity for eight reaction cycles when the catalyst consisting of carbon and strontium oxide was applied. In addition, the authors suggested the use of this catalyst in the transesterification reaction regardless of the oil used in the process.

Thus, the present study aims to synthesize a heterogeneous basic catalyst based on biochar produced from the residual biomass of murici functionalized with sodium. It also aims to evaluate its applicability in the transesterification reaction of soybean oil via the methyl route, as well as to study its ability to be reused and regenerated in the process of biodiesel synthesis.

## 2. Results and Discussion

### 2.1. Influence of Sodium Percentage

**Figure 1:**
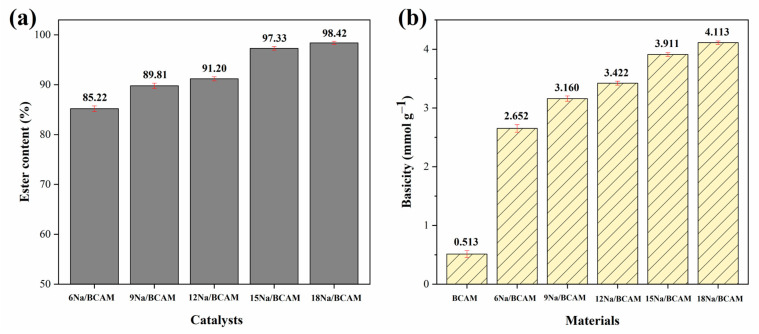
(**a**) Influence of the sodium percentage present in the catalysts and (**b**) basicity of BCAM support and catalysts synthesized.

One of the main factors that can influence the ester content of biodiesel, as well as its other physicochemical properties, is the catalyst used in the transesterification reaction and this, in turn, has a direct relationship with its synthesis process. Thus, the influence of sodium percentages 6, 9, 12, 15 and 18% on the final catalyst composition in the biodiesel production process was evaluated. In this preliminary step, the catalytic tests were performed under fixed reaction parameters: temperature of 120 °C, reaction time of 3 h, catalyst concentration of 8% and MeOH:oil molar ratio of 24:1. Figure 1 presents the results obtained in the study on the influence of the percentage of sodium present in the catalysts, as well as the basicity data of the murici biochar (BCAM) support and catalysts studied.

Based on Figure 1a, it is initially inferred that there is a direct relationship between the percentage of functionalization of biochar and the ester content of biodiesel. During the study, it was observed that 6Na/BCAM did not efficiently conduct the transesterification reaction due to providing the lowest ester content value in biodiesel (85.22% ± 0.55) among all catalysts used. The low catalytic activity presented may be related to the insufficient amount of active sites responsible for the conversion of triglycerides into esters [41,42]. In addition, it is possible to observe that by tripling the percentage of active phases present in the catalyst composition, that is, by employing the catalyst 18Na/BCAM, an increase of 13.20% in the ester content of biodiesel is observed, reaching the maximum value of 98.42% ± 0.29 and establishing a linear growth trend of the ester content of biodiesels as the percentage of the active phase impregnated in the BCAM support increases.

It is worth noting that the catalysts 15Na/BCAM and 18Na/BCAM present biodiesels with similar ester contents, with about a 1.09% difference. This may be an indication that impregnations with higher percentages of active phases would not cause a significant impact on the catalytic performance of the catalysts during the conduction of the transesterification reaction. In addition, this fact may be linked to the saturation of these active sites or the high viscosity of the reaction system together with the excess of the active phase, hindering the mass transfer between the MeOH:oil system with the active sites of the catalyst and leading to negligible increases in the conversion of triglycerides to esters [7,18]. Thus, considering the small difference between the synthesized biodiesels and the additional operational cost in the synthesis process to impregnate 3% more active phase in the BCAM support, the percentage corresponding to 15% sodium (the catalyst 15Na/BCAM) was selected for the continuity of the study.

In addition, the selected catalyst leads to a biodiesel with an ester content of 97.3%, 14 times higher than the ester content of biodiesel achieved when only the BCAM support was used (6.95% ± 0.36 ester content). The increase in ester conversion observed can be attributed to the increase in the basic groups of the catalyst 15Na/BCAM (3.911 mmol g^−1^ ± 0.031) relative to the BCAM support (0.513 mmol g^−1^ ± 0.060), caused by the process of impregnation of the active phase, as can be seen in the data presented in Figure 1b. This behavior was also observed by Zhao et al. [31], who reported basicity values of 0.2 mmol g^−1^ for the pomelo peel biochar and 9.0 mmol g^−1^ for the catalyst based on biochar impregnated with 25% K_2_CO_3_ achieving biodiesel with yields of 5.6% and 98%, respectively. Furthermore, in the study by Zhao et al. [30], the basicity value for rice husk biochar was 1.5 mmol g^−1^, while the basicity determined for the catalyst impregnated with 30% CaO was 11.4 mmol g^−1^, resulting in biodiesels with yields of 12.5 and 93.4%, respectively.

It is noteworthy that the 15Na/BCAM catalyst demonstrated high catalytic activity with 15% of the active phase impregnated on the support (ester content of 97.33% ± 0.38), while the avocado biochar catalyst functionalized with 20% Ca (*w*/*w*) led to a biodiesel with methyl ester content (FAME) of only 82.7% [29]. In addition, Zhang et al. [4] synthesized a magnetic catalyst Na_2_SiO_3_@Ni/C that was used in the optimal reaction condition, resulting in a biodiesel with a yield of 98.1%, a value slightly higher than the ester content obtained in this study when the catalyst 15Na/BCAM was used. However, this yield of 98.1% achieved in the use of the catalyst in the Na_2_SiO_3_@Ni/C is conditioned on the use of 56% sodium silicate, the equivalent of 21.1% sodium, that is, a 6% more active phase compared to that present in the catalyst 15Na/BCAM developed in this study.

### 2.2. Characterization of Materials

#### 2.2.1. Elemental Analysis (CHNS)

The results of the elemental analysis for murici seed, BCAM support and catalyst 15Na/BCAM are shown in Table 1. When comparing the results obtained for the murici seed biomass and for the BCAM support, it can be noticed that the carbon (C) content increases from 45.17 to 71.72%, while the oxygen (O) content decreases from 50.87 to 24.81%. This can be attributed to the thermal degradation of the lignocellulosic components during the carbonization process of the murici seed, which results in the elimination of groups that have oxygen, hydrogen, and nitrogen in their structure, resulting in an increase in the carbon content present in the BCAM support [36,43]. In addition, there is a subtle decrease in the hydrogen content (H) from 2.88 to 1.49% and nitrogen (N) from 2.82 to 1.82%. The results obtained for the catalyst 15Na/BCAM suggest an increase from 24.81 to 28.60% in the oxygen (O) content compared to the BCAM support and indicate the presence of silicon (Si) and sodium (Na) percentages of 9.53 and 5.82%, respectively. These elements are assigned to the impregnation step carried out in the catalyst synthesis process.

#### 2.2.2. TG/DTG Thermogravimetric Analysis

**Figure 2:**
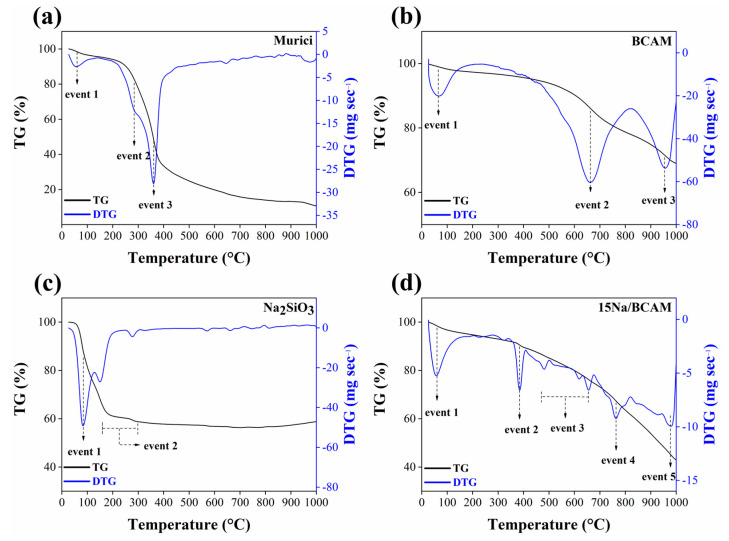
TG/DTG plots of (**a**) murici seed, (**b**) BCAM support, (**c**) Na_2_SiO_3_ and (**d**) catalyst 15Na/BCAM.

The knowledge of the thermal stability of the synthesized materials at different temperatures is of paramount importance since it helps in optimizing the process of preparing the materials [16]. Thus, TG and DTG thermogravimetric analyses were performed in order to understand the synthesis process of the BCAM support and the catalyst 15Na/BCAM. In addition, the thermal stability of the BCAM support after its formation was also analyzed, as well as the sodium silicate precursor (Na_2_SiO_3_), as can be seen in Figure 2.

Figure 2a presents the TG and DTG curves for the murici seed, in which the occurrence of three main mass loss events is observed. Event 1, corresponding to a mass loss of approximately 4%, occurs in the 25–100 °C range and can be attributed to the loss of water molecules adsorbed on the surface of the material [31]. Event 2, about 13.5%, temperature range 140–286 °C, is attributed to the onset of pyrolysis of lignocellulosic components of lower thermal stability, i.e., hemicellulose [43,44]. Event 3, with a mass loss of 29%, in the 200–380 °C temperature range, corresponds to the thermal degradation of the other lignocellulosic components, cellulose, and lignin, which have more complex structures and, as a consequence, are more resistant to pyrolysis [44].

From the analysis of the TG and DTG curves referring to the BCAM support, shown in Figure 2b, it is possible to identify three mass loss events. Event 1, approximately 2%, temperature range 25–100 °C, shows a loss of water physically bound to the surface [45], while the mass losses in the temperature range 430–640 °C (event 2) and between 910 and 980 °C (event 3) correspond to pyrolysis of lignocellulosic compounds (hemicellulose, cellulose, and lignin) not volatilized during biochar synthesis by carbonization of the murici seed [27].

Figure 2c,d show the TG and DTG curves for Na_2_SiO_3_ and catalyst 15Na/BCAM, respectively. The TG and DTG curves present in Figure 2c indicate the occurrence of two main mass loss events. The first, of 13.5% (range 25–85 °C), and the second, 29% (range 100–300 °C), can be attributed to the elimination of water molecules (i) adsorbed on the surface and (ii) structurally bound to the material [46,47]. Based on the analysis of the TG and DTG curves shown in Figure 2d, it is possible to identify five main events of mass loss. Event 1, which corresponds to the temperature range 58–100 °C, refers to a mass loss of 3% and may be attributed to loss of physically bound water and structure [45]. Event 2, in the temperature range 350–400 °C, corresponds to a mass loss of 3% and is related to the process of dihydroxylation of OH groups [31]. The mass loss event 3, with about 10% and occurring in the temperature range 470–650 °C, and event 4, with a mass loss of approximately 2% in the temperature range 740–765 °C, as well as event 5, with mass loss of 4.5% in the range 940–980 °C, can be attributed to the degradation of carbonaceous structures (hemicellulose, cellulose, and lignin) not completely carbonized during the preparation of the BCAM support [26,27,48].

#### 2.2.3. X-ray Diffraction (XRD)

**Figure 3:**
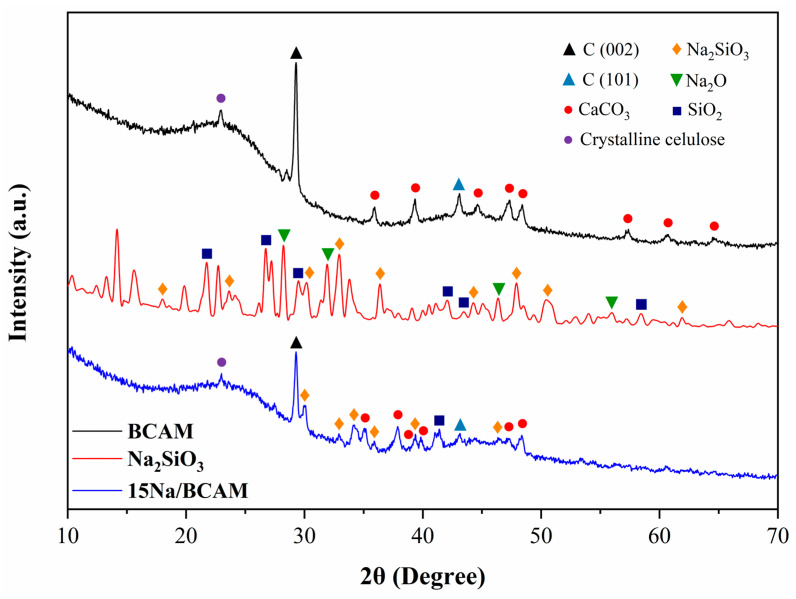
XRD patterns of BCAM support, Na_2_SiO_3_ and catalyst 15Na/BCAM.

X-ray diffractograms for the BCAM support, in the Na_2_SiO_3_ and catalyst 15Na/BCAM, are set forth in Figure 3. In the diffractogram referring to the BCAM support (black line), a diffraction peak characteristic of the presence of lignocellulosic materials in the region of 2θ = 22.5° associated with the microcrystalline structure of cellulose can be observed [12]. In addition, the diffractogram showed a halo in the region of 2θ = 15–30° assigned to plane 002 typical of amorphous carbon structures, and a less intense halo in the region of 2θ = 40–50° to plane 101 of amorphous carbonaceous structures [26,27], while the peaks at 2θ = 28.9° and 2θ = 43.14° correspond to planes 002 and 101 in graphitic carbons, respectively [49]. The diffractogram for the BCAM support showed peaks in regions 2θ = 36°, 39.3°, 44.6°, 47.3°, 48.4°, 57.34°, 61° and 65° attributed to the CaCO_3_ structure [50,51,52].

It is worth highlighting that the presence of inorganic materials in the biochar structure is reported in the literature, such as in the studies conducted by Jitjamnong et al. [32], who synthesized a biochar derived from banana peel and observed the presence of potassium and calcium oxides. Conversely, in their study with biochar from banana peel, Patel et al. [33] reported the presence of CaCO_3_ structures. In the studies reported by Zhao et al. [30], the presence of SiO_2_ was observed in the structure of biochar obtained from rice husk; they concluded that the presence of these inorganic structures does not attribute catalytic activity to biochar when applied in the transesterification reaction, for it achieved a biodiesel yield of only 12.5%.

Major peaks present in the diffractogram of Na_2_SiO_3_ (red line) in the regions of 2θ = 17.1°, 24°, 32.9°, 36.4°, 44.5°, 47.9°, 50.6° and 61.8° are mainly attributed to the structure of Na_2_SiO_3_.9H_2_O and crystalline Na_2_SiO_3_ in the orthorhombic phase [53,54]. In addition, the peaks present in 21.8°, 26.7°, 29.5°, 42.2°, 43.4° and 58.4° indicate the presence of SiO_2_ [8,42,55,56]. The presence of Na_2_O is indicated by peaks in the regions of 28.3°, 32°, 46.3° and 55.9° [57]. In the diffractogram relative to the catalyst 15Na/BCAM (blue line), the peak was initially observed in 2θ = 22.5° assigned to the microcrystalline phase of cellulose [12], and peaks at 2θ = 28.9 and 2θ = 43.14 were assigned to planes 002 and 101 in graphitic carbons, respectively [49]. In addition, the typical halos of amorphous carbonaceous materials were observed in the regions of 2θ = 15–30° and 2θ = 40–50° assigned to plane 002 and plane 101, respectively [26,27]. Finally, peaks were observed in 2θ = 35.1°, 37.8°, 38.8°, 39.84°, 47.1° and 48.4°, attributed to the CaCO_3_ structure [50,51] and at 2θ = 30.1°, 32.9°, 34.12°, 35.9°, 39.4° and 46.4°, characteristic of the orthorhombic crystalline phase of Na_2_SiO_3_ [53,54].

#### 2.2.4. Fourier Transform Infrared Spectroscopy (FT-IR)

**Figure 4:**
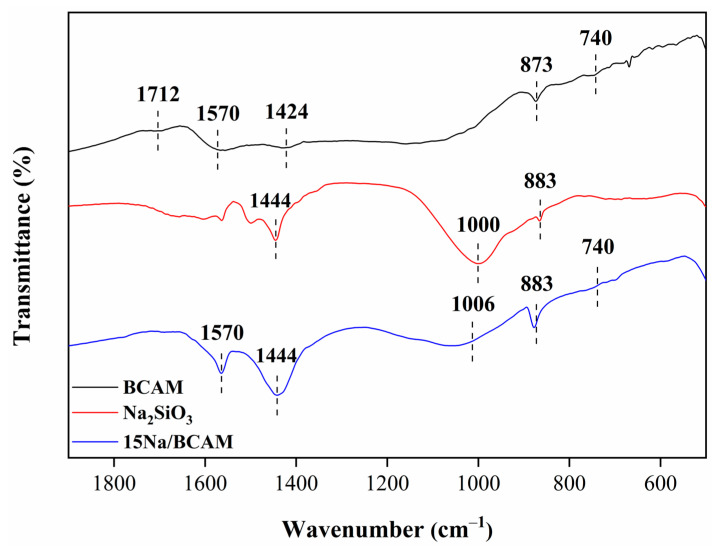
FT-IR spectra of BCAM support, Na_2_SiO_3_ and catalyst 15Na/BCAM.

FT-IR spectra for the BCAM support in the Na_2_SiO_3_ and catalyst 15Na/BCAM are set forth in Figure 4. For the spectrum of the BCAM support (black line), typical vibrational bands of carbonized lignocellulosic materials were identified at 1712 and 1570 cm^−1,^ attributed to the C=O and C=C bond stretch, respectively, characteristics of the presence of carboxylic groups and aromatic rings [12,26]. The vibrational bands observed at 1424 cm^−1^ and 873 cm^−1^ correspond to antisymmetric stretching and out-of-plane and in-plane vibrational modes, respectively, assigned to the carbonate group (CO^−2^) [33]. This reinforces the presence of CaCO_3_ structures, as indicated by the XRD analysis. In addition, the presence of the vibrational band in the region of 740 cm^−1^ is observed in the spectrum assigned to bond stretch =C–H [26].

The spectrum referring to the Na_2_SiO_3_ (red line) shows vibrational bands at 1444 cm^−1^ and 1000 cm^−1^ characteristics of symmetric and antisymmetric stretches of Si–O–Si and Si–O–Na bonds, respectively [53,58]. The vibrational band present at 883 cm^−1^ can be attributed to the antisymmetric stretching of the bond Si–O–H characteristic of bonds of silanol groups present in the hydrated form of sodium silicate (Na_2_SiO_3_·9H_2_O) [58]. Finally, the spectrum referring to the catalyst 15Na/BCAM (blue line) shows vibrational bands in the regions of 1570 cm^−1^ and 740 cm^−1^ assigned to C=C and =C–H bond stretches, respectively, characteristics of aromatic chains [12,26]. The vibrational bands present at 1444 cm^−1^ and at ~1006 cm^−1^ are attributed to the antisymmetric stretching of the bonds Si–O–Si and antisymmetric Si–O–Na, respectively [53,58]. In addition, a vibrational band is observed in the region of 883 cm^−1^ attributed to the antisymmetric stretching of the Si–O–H bond [58].

#### 2.2.5. Scanning Electron Microscopy (SEM)

**Figure 5:**
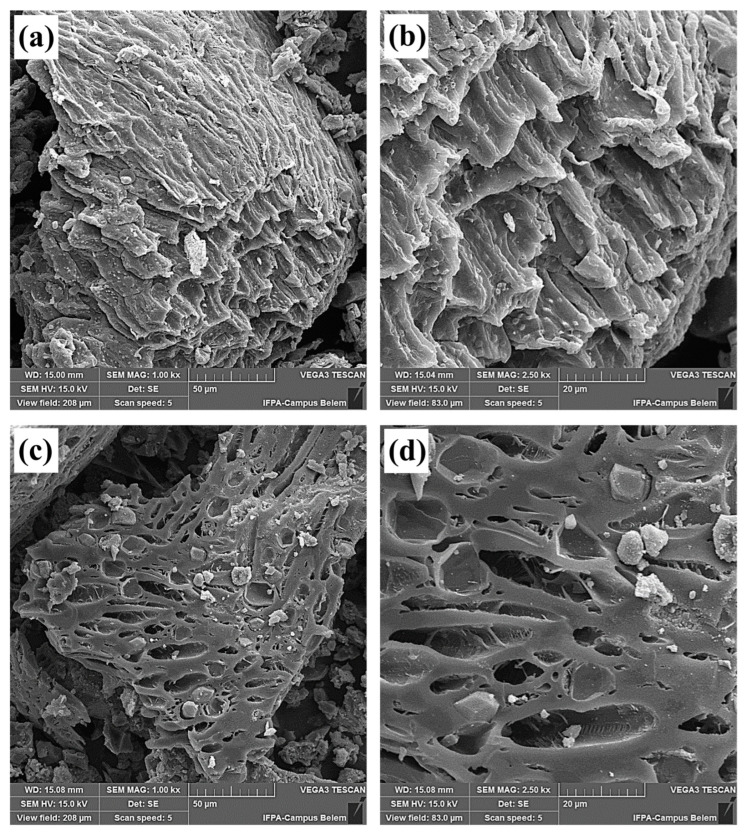
SEM micrographs (**a**) BCAM support 1000× magnification, (**b**) BCAM support 2500× magnification, (**c**) catalyst 15Na/BCAM 1000× magnification and (**d**) catalyst15Na/BCAM 2500× magnification.

Micrographs of the BCAM support and the catalyst 15Na/BCAM are shown in Figure 5. The micrographs referring to the BCAM support are shown in Figure 5a,b and indicate that the BCAM support has an irregular surface morphology with a wide well-developed pore network, formed by the release of gases during the carbonization of organic matter rich in lignocellulosic compounds [26,29,37]. Figure 5c,d show the surface morphology of the catalyst 15Na/BCAM, in which it is possible to observe a marked decrease in the porosity of the support. This is directly related to the impregnation process and, as a consequence, of the anchoring of sodium silicate on the biochar surface. In addition, it can be seen that the deposited sodium silicate does not possess a defined morphology, with small, agglomerated regions of whitish color [46,59].

#### 2.2.6. Energy Dispersion X-ray Spectroscopy (EDS)

**Figure 6:**
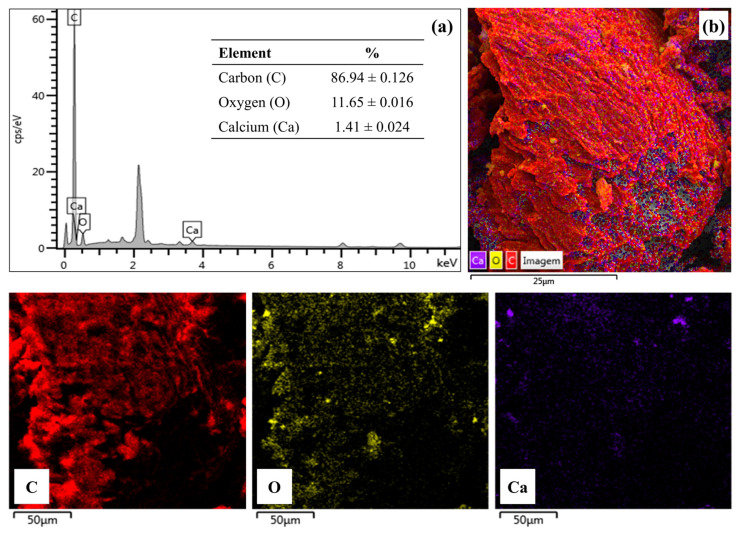
EDS (**a**) chemical composition and (**b**) elementary mapping of the chemical elements on the surface of BCAM support.

The composition and elemental mapping for the BCAM support are shown in Figure 6. It can be observed through the composition (Figure 6a) and elemental mapping (Figure 6b) that the BCAM support has a large carbon content (C), about 86.94% ± 0.126 distributed homogeneously, and a relatively low oxygen content (O), approximately 11.65% ± 0.016, homogeneously dispersed in the material with small concentration regions. Both are directly related to the pyrolysis process in nitrogen atmosphere to which the biomass was subjected. Furthermore, these values are close to those indicated in the results of CNHS, 71.72% ± 0.03 for the carbon content (C) and 24.76% for the oxygen content (O). This small difference between the contents found may be linked to the method of quantification of the elemental composition of the sample used by each technique. Additionally, the composition (Figure 6a) and the elemental mapping (Figure 6b) for the BCAM support indicated the presence of small regions containing calcium (Ca) and oxygen (O) homogeneously and in small clusters. These results are in consensus with the results shown in the XRD analysis, which indicated the presence of calcium carbonate in the crystalline phase present in the BCAM support. However, despite constituting about 1.41% ± 0.024 of the sample, this percentage of calcium carbonate does not attribute catalytic activity to the BCAM support since the ester content value achieved for the biodiesel produced from the reaction using only the BCAM support (blank reaction) was 6.95% ± 0.36.

**Figure 7:**
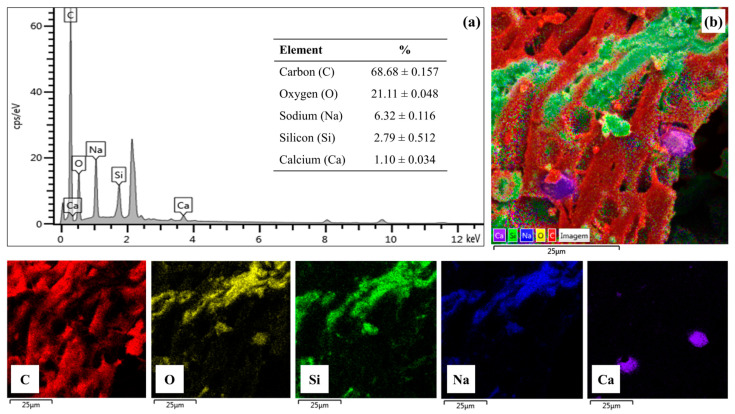
EDS (**a**) elementary composition and (**b**) elemental maps of each constituent of the catalyst 15Na/BCAM.

Figure 7 shows the composition and elemental mapping for catalyst 15Na/BCAM. The elemental composition shown in Figure 7a initially exposes the presence of 6.32% ± 0.157 sodium in the catalyst, which suggests that the impregnation process was efficient. In addition, the presence of 2.79% ± 0.512 silicon (Si) is also observed, a value close to the value of the 2:1 stoichiometric ratio of Na:Si present in Na_2_SiO_3_. Moreover, it is possible to perceive the presence of the elements carbon (C), oxygen (O), and calcium (Ca) in the values of 68.68% ± 0.157, 21.11% ± 0.048 and 1.1% ± 0.034, respectively, referring to the elemental composition of the BCAM support. It should be noted that the increase in the percentage of the oxygen element (O) is directly related to the process of impregnation of the BCAM support as a function of the percentage of sodium from the precursor in the Na_2_SiO_3_. Additionally, the elemental mapping for the 15Na/BCAM catalyst shown in Figure 7b suggests good dispersion of the elements carbon (C), oxygen (O), sodium (Na), silicon (Si), and calcium (Ca) on the catalyst surface.

### 2.3. Study of the Influence of Reaction Parameters for Biodiesel Synthesis

**Figure 8:**
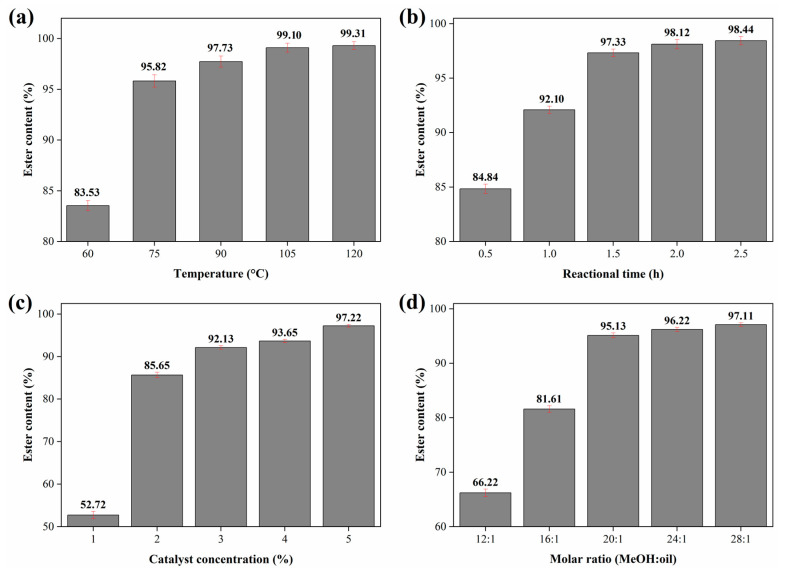
Influence of reactional parameters (**a**) temperature, (**b**) reaction time, (**c**) catalyst concentration and (**d**) MeOH:oil molar ratio in the process of production biodiesel using the catalyst 15Na/BCAM.

The study of process optimization is one of the main aspects that must be considered so that the biodiesel production process becomes economically viable for large-scale commercialization [16]. Thus, in order to determine the optimal reaction conditions, the study of the reaction variables temperature, reaction time, catalyst concentration and MeOH:oil molar ratio in the transesterification reaction of soybean oil using the catalyst 15Na/BCAM was performed. Figure 8 shows the results obtained.

The temperature parameter is one of the factors with the greatest influence on the transesterification reaction. The study range used for temperature was 60–120 °C, keeping the other variables fixed: reaction time of 2 h, catalyst concentration 3% (*w*/*w*) and MeOH:oil molar ratio of 20:1. It can be inferred from Figure 8a that, as the reaction temperature rises, there is an increase in the conversion of triglycerides into esters, as observed at the reaction temperatures of 60, 75, 90, 105 and 120 °C, in which the synthesized biodiesels obtained ester contents of 83.53 ± 0.51, 95.82 ± 0.61, 97.73 ± 0.55, 99.10 ± 0.44 and 99.31% ± 0.51, respectively. Initially, this may be related to the endothermic character of the transesterification reaction, which requires quantities of heat to proceed in a straight direction [56], that is, as the temperature increases, there is a tendency to produce biodiesels with higher ester contents. In addition, high temperatures in the process promote better interactions between the reagents and, as a consequence, there are greater interactions of these with the active sites of the catalyst, resulting in better conversions [41]. However, it was observed that when the temperature was raised from 105 to 120 °C and from 90 to 105 °C, there were small increases in the ester contents of biodiesels, by about 0.21 and 1.58%, respectively. It is worth noting that the temperatures of 75 and 90 °C led to biodiesels with similar ester contents with a difference of only 1.91%. Thus, considering the energy cost to provide an additional 15 °C, the temperature of 75 °C was selected for the continuity of the study.

The reaction time is also an essential parameter when considering the biodiesel production process, as it significantly influences the catalytic activity of the catalyst used in the process [7]. The time optimization study was performed in the range of 0.5–2.5 h, fixing the other variables: temperature 75 °C, catalyst concentration 3% (*w*/*w*) and MeOH:oil molar ratio of 20:1. The data illustrated in Figure 8b suggest that the reaction time of 0.5 h is insufficient to conduct the reaction efficiently, providing a biodiesel with an ester content of 84.84% ± 0.42. This fact can be attributed to insufficient time for the interaction between the reagents and the active sites of the catalyst [42]. It is also possible to suggest, based on Figure 8b, a linear growth relationship between the reaction time and the ester content, as observed more explicitly in the reaction times of 0.5, 1.0 and 1.5 h, which led to biodiesels with ester contents of 84.84 ± 0.42, 92.10 ± 0.33 and 97.33% ± 0.34, respectively. However, when the reaction time was increased from 2 h to 2.5 h, a subtle increase in the ester content of about 0.32% was observed. In addition, the reactions conducted in the reaction times of 1.5 and 2 h provided biodiesels with very close ester contents, with a difference of 1.11%. Thus, in view of the economy of the biodiesel production process, the reaction time chosen was 1.5 h.

The study on the influence of catalyst concentration is illustrated in Figure 8c. The variables temperature of 75 °C, reaction time of 1.5 h and MeOH:oil molar ratio of 20:1 were kept fixed and the concentration of catalyst used in the transesterification reaction varied from 1 to 5%. When the reaction was processed using a 1% catalyst concentration, a biodiesel with ester content of 52.72% ± 0.84 was obtained. This low conversion to esters observed is directly linked to the insufficient number of active sites available for the reaction to process efficiently, as reported by Jamil et al. [21]. In addition, it was observed that, as the concentration of catalysts used in the reaction increased, there was an increase in the content of esters in biodiesels, reaching a maximum value of 97.22% ± 0.31 when the concentration of 5% was used in the process. Thus, the catalyst concentration of greater viability for the reaction process was 5% (*w*/*w*).

Finally, the influence of the MeOH:oil molar ratio on the transesterification reaction is shown in Figure 8d. The parameters of temperature 75 °C, reaction time 1.5 h and catalyst concentration of 5% (*w*/*w*) defined during the study were kept fixed. Numerous studies based on the stoichiometry of the transesterification reaction report that only 3 moles of alcohol are needed for the reaction to proceed. However, based on the reversible character of this reaction, excess alcohol is used [21]. It can be inferred from Figure 8d that the biodiesels obtained from the molar ratios of 12:1 and 16:1 presented low ester contents of 66.22 ± 0.68 and 81.61% ± 0.61, respectively. This trend may be related to an insufficient amount of alcohol in order to react with the triacylglycerols [42]. However, when the molar ratio was raised from 24:1 to 28:1, there was a small increase in conversion, about 0.89%. This may be attributed to the extremely diluted reaction system caused by excess methanol, which hinders the interaction between the reagents and the active sites of the catalyst [26]. It is worth highlighting that, when using the MeOH:oil molar ratio of 20:1, a biodiesel with an ester content of 95.13% ± 0.45 was obtained, a value very close to the ester contents of 96.22 ± 0.38 and 97.11% ± 0.35 of the biodiesels synthesized using the molar ratios of 24:1 and 28:1, respectively. Thus, the MeOH:oil molar ratio of 20:1 proved to be ideal and economically viable for the reaction process studied.

### 2.4. Physical and Chemical Properties of Biodiesel

The biodiesel synthesized using the catalyst 15Na/BCAM under optimal reaction conditions was characterized physicochemically according to the ASTM D6751 norm [60], with the results as shown in Table 2. It is observed that the values of 4.47 mm^2^ s^−1^ and 0.880 g cm^−3^ regarding the kinematic viscosity and density of biodiesel, respectively, comprise the limits stipulated by ASTM D6751. Such values of these properties for biodiesel are of paramount importance since they are directly related to the fuel flow capacity and atomization during fuel injection into the engine [15,21]. The biodiesel obtained during the study is in accordance with the limit established by the ASTM D6751 norm, since the acidity value determined was 0.2 mg KOH g^−1^. This data suggest a lower formation of scale in the engine components, since relatively small acidity values in biodiesel lead to an increase in the service life of pumps and filters [21,49].

The cold plugging point is directly related to the amount of esters derived from long-chain saturated fatty acids, which can lead to clogging of engine filters during fuel cooling [8,67]. The observed value for the cold plugging point was 0.0 °C, indicating that the biodiesel synthesized in this study can be used in engines, even in regions of low temperatures. The flash point is the main aspect responsible for certifying the safety of a fuel because it indicates the amount of residual methanol present in the biodiesel [17,49]. The value obtained for the synthesized soybean biodiesel was 150 °C, a value very similar to that reported by Mares et al. [8], which was 152 °C for biodiesel obtained using the ASA-800/4 catalyst under optimal reaction conditions. The value determined for the copper corrosivity parameter was 1a, which is within the limit stipulated by the ASTM D6751 norm. Thus, the data explained testify that the catalyst 15Na/BCAM is suitable for the synthesis of biodiesel since such a biofuel demonstrates high quality and compliance with the ASTM D6751 norm.

### 2.5. Catalyst Reuse Study

**Figure 9:**
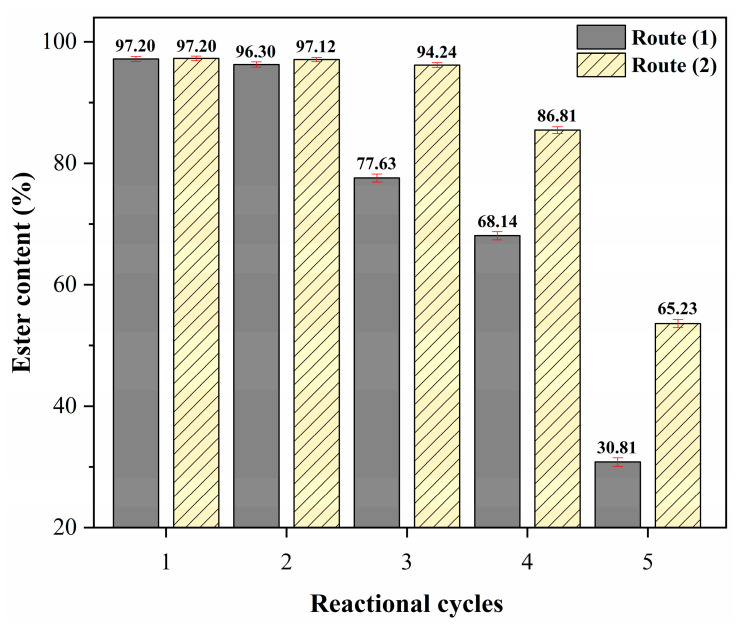
Study of reuse cycles of the catalyst 15Na/BCAM recovered using Route (1) and Route (2). Reaction conditions: temperature of 75 °C, reaction time of 1.5 h, catalyst concentration of 5% (*w*/*w*) and MeOH:oil molar ratio of 20:1.

Among the numerous advantages offered by the application of heterogeneous catalysts in the transesterification of oils, the ability to reuse and the possibility of regeneration are indispensable characteristics when considering the reduction of biodiesel production costs [12]. The reuse study of the catalyst 15Na/BCAM over several reaction cycles using the optimal reaction conditions, temperature of 75 °C, reaction time of 1.5 h, catalyst concentration of 5% (*w*/*w*) and MeOH:oil molar ratio of 20:1, is shown in Figure 9.

From the data shown in the graph of Figure 9, it is possible to observe that the catalyst 15Na/BCAM remained effective in its second reaction cycle because the biodiesel presented an ester content of 96.30% ± 0.42, a decrease of only 0.9% in relation to the biodiesel produced in the first reaction cycle, which was 97.20% ± 0.41. In addition, it can be inferred that the active sites present in the synthesized catalyst were not easily leached into the reaction medium since the biodiesels obtained in the third and fourth reaction cycles showed a reduction in ester content of 19.57 and 29.06%, respectively, in relation to the initial reaction. However, the fifth reaction cycle showed a significant decrease in catalytic activity, providing a product with an ester content of 30.81% ± 0.71. This decrease in ester content may be related to the deposition of unconverted mono-, di-, triglycerides, residual glycerol or esters not removed during the recovery process using Route (1), which consists only of washing with solvents (ethyl alcohol + hexane). This leads to the blocking of active sites on the surface of the catalyst, which makes it difficult for the reaction to proceed efficiently [68].

This hypothesis was tested by performing Route (2), which consists of the combination of the solvent washing process and the thermal reactivation of the catalyst in the tubular oven at 400 °C for 1 h in an atmosphere of N_2_ after each reaction cycle. Based on the data shown in Figure 9, it can be inferred that the hypothesis raised is true since the catalyst 15Na/BCAM recovered using Route (2) showed a better catalytic performance throughout the reuse cycles studied, that is, greater stability in the reaction medium since the biodiesels synthesized in the second, third, and fourth reaction cycles resulted in ester contents of 97.12 ± 0.35, 94.24 ± 0.38 and 86.81% ± 0.55, respectively. The biodiesel obtained in the fifth reaction cycle showed a reduction of 31.97% compared to the initial reaction, resulting in an ester content of 65.23% ± 0.65.

This downward trend in the performance of the catalyst 15Na/BCAM may be related to leaching of a significant amount of basic sites. Table 3 shows the results of the basicity analysis of the fresh catalyst, that is, before being used in the reaction and of the catalyst used after the fifth reaction cycle recovered via Route (2). It is observed in Table 3 that there was a decrease in the presence of basic groups between the fresh and the recovered catalysts via Route (2), since they presented basicity values of 3.911 mmol g^−1^ ± 0.031 and 1.420 mmol g^−1^ ± 0.011, respectively. In addition, the data shown in Figure S1 (available in the Supplementary Materials) reinforce the data shown about the basicity of the catalysts, since the percentage of sodium in the catalyst after the fifth reaction cycle was 1.84% ± 0.056, while in the catalyst before the reaction it was 6.32% ± 0.116. This behavior was also reported in the study developed by Zhao et al. [30], in which the authors observed a decrease in catalytic activity between the catalyst used in the first use and the catalyst used in the eighth reaction cycle, which provided biodiesels with yields of 98% and 82.4%, respectively. In addition, the authors justify these results by the decrease in basicity over the reuse cycles since the basicity value for the fresh catalyst was 9.0 mmol g^−1^, while the basicity determined for the catalyst after the 8° reaction cycle was 4.8 mmol g^−1^.

### 2.6. Catalyst Regeneration Study

**Figure 10:**
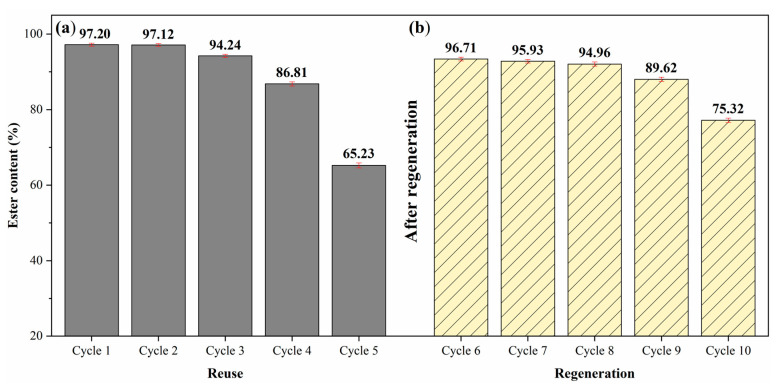
(**a**) Reuse study of the catalyst 15Na/BCAM recovered using Route (2) and (**b**) study of regeneration of the catalyst 15Na/BCAM. Reaction conditions: temperature of 75 °C, reaction time of 1.5 h, catalyst concentration of de 5% (*w*/*w*) and MeOH:oil molar ratio of 20:1.

During the reuse study of the catalyst 15Na/BCAM recovered via Route (2), a decrease in the ester content of biodiesel produced in the fifth reaction cycle of about 31.97% compared to the first reaction cycle was observed. In general, this reduction in catalyst performance can be attributed to a decrease in the basic active sites responsible for conducting the transesterification reaction efficiently. Given this, the applicability of the catalyst regeneration process using sodium silicate in an amount that would allow obtaining the concentration of 10% (*w*/*w*) of sodium was evaluated in the final composition of the catalyst. Figure 10 illustrates the data obtained in the study of the catalyst regeneration process.

Figure 10a presents the data corresponding to the reuse study of the catalyst 15Na/BCAM recovered Route (2), the starting point for the development of the study of the regeneration process. From the data presented in Figure 10b, it is possible to infer that the catalyst regeneration process was successful since the biodiesel ester content of 96.71% ± 0.65 obtained in the sixth reaction cycle (first after regeneration) represents an increase of 31.48% in relation to the fifth reaction cycle. In addition, the catalyst used in the seventh and eighth reaction cycles led to biodiesels with ester contents of 95.93 ± 0.63 and 94.96% ± 0.73, respectively, which represents insignificant decreases of 0.78 and 1.75% in the ester contents of the biodiesels produced when compared to the sixth reaction cycle. The data suggest that the catalyst 15Na/BCAM exhibits excellent catalytic stability in the reaction medium. However, after the ninth reaction cycle of the catalyst, a decrease in catalytic activity was observed, since the biodiesels synthesized in the ninth and tenth reaction cycles obtained ester contents of 89.62% ± 0.76 and 75.32% ± 0.77, respectively, representing a reduction in catalyst performance by about 7.09 and 21.39% compared to the first use after regeneration process (sixth reaction cycle), as shown in Figure 10b.

The applicability and efficiency and reusability of the catalyst 15Na/BCAM regenerated with 10% (*w*/*w*) sodium and recovered via Route (2) in the conduction of the transesterification reaction are highlighted due to the fact that it produces biodiesels with ester contents above 89% in the ninth reaction cycle. In addition, the catalyst 15Na/BCAM is highly stable in the reaction medium when compared to the acai seed ash catalyst (ASA-800/4), also submitted to the regeneration study using 20% (*w*/*w*) of KOH, which showed a decline in its seventh reaction cycle (third reaction cycle after regeneration) resulting in a biodiesel with ester content of 84.9%, and at the end of the ninth reaction cycle (fifth reaction cycle after regeneration) showed significant loss in catalytic activity reaching an ester content of only 36.7% [8], while the catalyst 15Na/BCAM regenerated with 10% (*w*/*w*) sodium and recovered via Route (2) in its tenth reaction cycle, maintains its catalytic activity producing a biodiesel with an ester content of above 75%.

### 2.7. Comparison of Catalyst 15Na/BCAM with Different Biochar-Based Catalysts

Table 4 illustrates the performance in the biodiesel production process of the catalyst 15Na/BCAM developed in this study, as well as other heterogeneous basic catalysts based on biochar from different biomasses reported in the literature. Based on the data presented, it can be inferred that the BCAM support has milder synthesis (carbonization) conditions since it is prepared at 600 °C per 1 h when compared to avocado seed biochar and rice husk, which are produced at 900 °C for 2 h and 700 °C for 3 h, respectively [29,30]. In addition, the heat treatment process to which the catalyst 15Na/BCAM was subjected, a temperature of 400 °C for 1 h, which is a lower parameter than the other catalysts presented in Table 4, can be highlighted.

Considering the reaction parameters for the synthesis of biodiesel in which the catalysts listed in Table 4 are used, it is possible to note some similarity with the reaction conditions used for the catalyst 15Na/BCAM. However, the catalyst 15Na/BCAM showed greater catalytic stability throughout the reaction cycles since it provided 10 reaction cycles (five before the regeneration process and five after the process) when used under optimal reaction conditions. This is evidenced when this value is compared with the stability presented by the catalysts 20 wt% Ca loaded and 30K/BP-600, which provided only three and five reaction cycles, respectively [29,32]. In addition, Table 4 shows that the 15Na/BCAM catalyst studied has good catalytic activity, resulting in biodiesel with an ester content of 97.20% ± 0.31 under mild reaction conditions (75 °C, 1.5 h, 5% and 20:1), compared to the catalytic performances of the CaO-MgO-800-5 and CaO/AC catalysts, which produce biodiesels with ester contents of 91.1% and 80.98%, respectively, under relatively abrupt reaction conditions of 190 °C, 1.35 h, 5.5% and 15:1 for the CaO-MgO-800-5 catalyst and 70 °C, 4.4 h, 8% and 21:1 for the CaO/AC catalyst [69,70]. Thus, the data listed in Table 4 reinforce the feasibility of using the residual biomass of the murici seed in the preparation of an efficient basic heterogeneous catalyst for the synthesis of biodiesel.

## 3. Materials and Methods

### 3.1. Materials

Murici fruit and refined soybean oil were purchased at a local market in the city of Belém, Pará, Brazil. Pure sodium silicate (Na_2_SiO_3_) (VERTEC^®^, Silchester, UK, 1344-09-8) was used in the process of biochar functionalization and catalyst regeneration. Sodium hydroxide (NaOH) 98.0% (Neon^®^, New York, NY, USA, 310-73-2) and hydrochloric acid (HCl) 37.0% (Qhemis^®^, Jundiaí, Brazil, 7647-01-0) were used in determining the basic groups present in murici biochar and catalyst. N-heptane UV/HPLC (C_7_H_16_) 99.0% (Neon^®^, 142-82-5) and methyl heptadecanoate (C_18_H_36_O_2_) 99.0% (Sigma-Aldrich^®^, St. Louis, MO, USA, 1731-92-6) were used in the quantification of esters by gas chromatography. Methyl alcohol (CH_3_OH) 99.8% (ÊXODO CIENTÍFICA^®^, Sumaré, Brazil, 67-56-1) was used in the transesterification reactions, while ethyl alcohol (C_2_H_5_OH) (Dinâmica^®^, Barcelona, Spain, 64-17-5) and n-hexane (C_6_H_14_) 95.0% (Neon^®^, 110-54-3) were used in catalyst recovery.

### 3.2. Catalyst Synthesis

**Figure 11:**
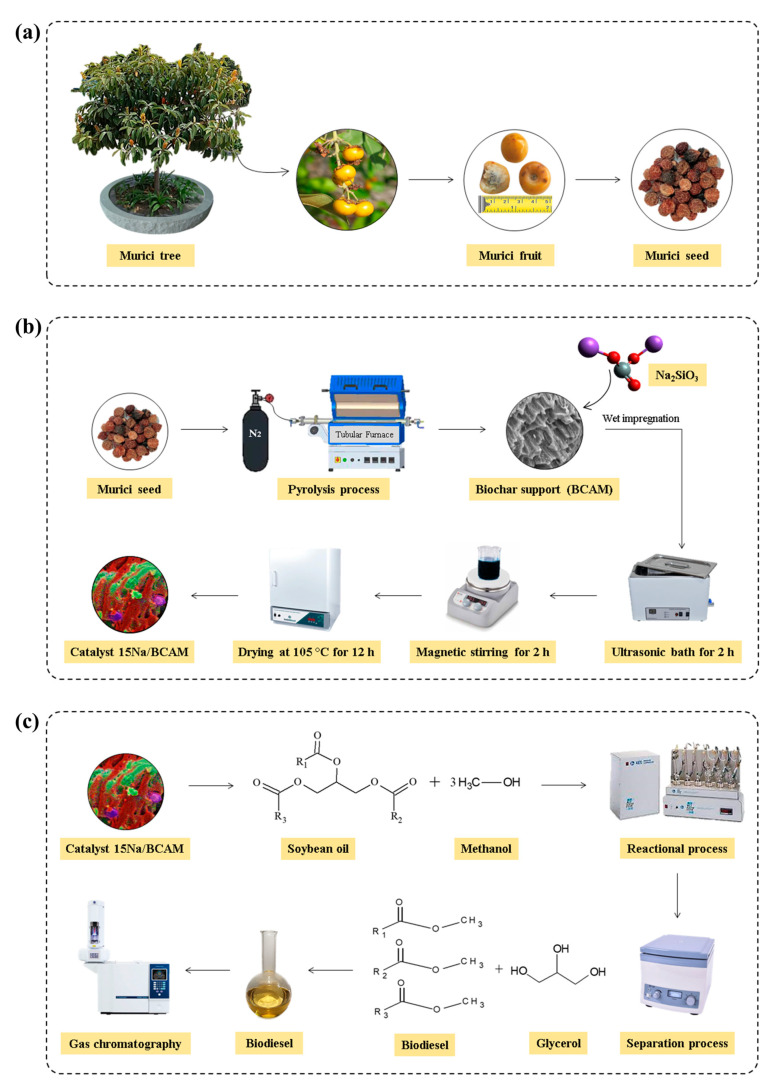
Scheme of synthesis (**a**) BCAM support, (**b**) catalysts xNa/BCAM and (**c**) biodiesel.

Initially, the murici fruit (Figure 11a) was pulped and washed with distilled water to remove dirt and remnants of the pulp. Then, the seeds were dried in an oven at 105 °C for 12 h. Then, the seeds were ground and sieved (10 mesh). Soon after, the material with higher granulometry (>10 mesh) was pyrolyzed in a tubular oven at 600 °C for 1 h with N_2_ flow of 80 mL min^−1^ according to the methodology described by Corrêa et al. [26], with minor modifications. Finally, the murici biochar (BCAM) was cooled to room temperature, packed in a hermetically sealed container and stored in a desiccator.

The preparation of catalysts based on sodium functionalized biochar was performed by wet impregnation according to the procedures described by Alsharif et al. [71] and Bitonto et al. [29], with adaptations, as shown in Figure 11b. Initially, the BCAM support mass was dispersed in a beaker with distilled water, to which the sodium silicate mass was added in amounts that made it possible to obtain the concentrations 6, 9, 12, 15 and 18% (*w*/*w*) of sodium in the final catalyst composition. In a typical process for the preparation of a catalyst impregnated with 15% (*w*/*w*) sodium, the mass of 5.0 g of dry and previously activated BCAM support at 105 °C was dispersed in 60 mL of distilled water and then was added to the mass of 3.35 g of sodium silicate. Then, the suspension was kept in an ultrasonic bath at 25 °C for 2 h, followed by magnetic stirring for 2 h. When it was finished, the mixture was dried in an oven at 105 °C for 12 h, followed by heat treatment in a tubular oven at 400 °C for 1 h under a flow of 80 mL min^−1^ of N_2_. The synthesized catalysts were named xNa/BCAM, in which x represents the impregnated sodium concentration.

### 3.3. Catalyst Characterization

The determination of the basic groups present in the biochar, catalyst and regenerated catalyst was performed according to the Boehm [72] method, with adaptations. In this method, a mass of 0.25 g of the material (BCAM support and catalyst xNa/BCAM) was dispersed in 30 mL of standard solution HCl 0.10 mol L^−1^ and kept being stirred for 24 h at room temperature. When it was finished, the mixture was centrifuged and an aliquot of 10 mL of the filtrate was mixed with 15 mL of standard NaOH solution 0.10 mol L^−1^. Then, the excess NaOH was titrated with HCl 0.10 mol L^−1^ solution, using phenolphthalein as an indicator. The Elemental Composition (CHNS) of the materials (murici seed, BCAM support, and xNa/BCAM) was performed on a Fisons Instruments EA1108 equipment with the aid of the EAGER 200-RESULTS software. The masses of approximately 2.0 mg of samples were weighed on a Sartorius microscale (XM-1000 P). Thermogravimetric Analysis (TG/DTG) for the murici seed, BCAM support, Na_2_SiO_3_, and xNa/BCAM was performed using Shimadzu equipment, model DTG-60H, between the temperature range of 25 °C to 1000 °C (heating rate of 10 °C min^−1^), in a flow of N_2_ of 50 mL min^−1^, using an alumina crucible. The X-ray Diffraction (XRD) patterns of the BCAM support, in the Na_2_SiO_3_ and xNa/BCAM, were obtained using the powder method in a PANalytical diffractometer, EMPYREAN model, with radiation employed and scanning range of Cu Kα (1.541874 Å) at 40 KV and 30 mA and 10° < 2θ < 70°, respectively. The analysis of Fourier Transform Infrared Spectroscopy (FT-IR) for the BCAM support, Na_2_SiO_3_, and xNa/BCAM, was performed using a Shimadzu spectrometer model IRPrestige-21 in the spectral range of analysis of 1900–500 cm^−1^ with 4 cm^−1^ resolution and 32 scans. The surface morphology of the BCAM support and xNa/BCAM was determined by Scanning Electron Microscopy (SEM) using a TESCAN microscope, model VEGA 3 LMU, operating with acceleration voltage of 20 kV. The elemental composition of the BCAM support and xNa/BCAM surface was determined by X-ray Spectroscopy by Energy Dispersion (EDS) using an Oxford microanalysis system, model AZTec Energy X-Act, with resolution of 129 eV.

### 3.4. Transesterification Reaction

The catalytic tests of methyl transesterification of soybean oil, using functionalized biochar as a catalyst, was conducted in a 80 mL capacity closed steel reactor of the Parr Series 5000 Multiple Reactor System under fixed agitation of 700 rpm. The reaction parameters were optimized from the evaluation of reaction temperature (60–120 °C), reaction time (0.5–2.5 h), catalyst concentration (1–5%), and MeOH:oil molar ratio (12:1–28:1). In a typical reaction procedure, 12 g of soybean oil was added to the reaction vessel, followed by the addition of 11.25 mL of methanol (referring to a MeOH:oil molar ratio of 20:1) and 0.6 g of catalyst (considering a catalyst concentration of 5%). Then, temperature and reaction time were set to 75 °C and 1.5 h, respectively. The catalyst concentrations used in catalytic tests were calculated as a function of the mass of oil employed in the reaction process.

At the end of each reaction experiment, the products were centrifuged to recover the catalyst, transferred to a settling funnel, and washed with portions of hot distilled water ~80 °C. Later, the remnants of washing water present were removed by drying in an oven at 60 °C for 12 h. Finally, the synthesized biodiesel was stored for the ester content analysis. A test was also performed using only the BCAM support (control) in order to evaluate its contribution to the catalytic activity of the catalyst in the reaction process. The biodiesel synthesis procedure is illustrated in Figure 11c.

### 3.5. Characterization of Biodiesel

#### 3.5.1. Ester Content

The determination of the ester content of biodiesels was performed according to the methodology adapted by Silva et al. [73] from the European standard EN 14103, using a Varian gas chromatograph (GC), model CP 3800, equipped with flame ionization detector (FID), capillary column CP WAX 52 CB (30 m length, 0.32 mm diameter and 0.25 µm film), with initial oven temperature programming of 170 °C, with heating rate of 10 °C min^−1^ up to 250 °C (same temperature as FID and injector). Helium gas was used in the flow of 1 mL min^−1^ as mobile phase, heptane as solvent, methyl heptadecanoate as internal standard, and an injection volume of 1.0 µL. The ester content (EC) was calculated using Equation (1).

(1) $$Ester\;content(\%)=\frac{\sum A_{T}-A_{P.I}}{A_{P.I}}\times \frac{C_{P.I}}{C_{B100}}\times 100$$

where: ∑*A_T_* is the sum of the total area of the ester peaks; *A_P.I_* is the area of the internal pattern; *C_P.I_* is the concentration of the internal standard solution (mg L^−1^); and *C_B_*_100_ is the final concentration of biodiesel after dilution (mg L^−1^).

#### 3.5.2. Physicochemical Properties of Biodiesel

The biodiesel produced was analyzed physicochemically using the American Society for Testing and Materials (ASTM) standard methodology [60]. The kinematic viscosity at 40 °C was determined according to ASTM D445 method [61], using a cannon-Fenske viscometer (SCHOTT GERATE, Model No. 520 23). The density was performed at 20 °C using the ASTM D1298 method [62], with an automatic KEM DAS-500 densimeter. The acid value of biodiesel was determined according to the ASTM D664 method [63]. The cold filter plugging point was determined according to the ASTM D6371 method [64] in a TANAKA equipment, model AFP-102. The flash point was determined according to the ASTM D93 method [65] on a TANAKA APM 7 Pensky-Martens automatic equipment. Finally, the corrosiveness of copper was determined according to the ASTM D130 method [66] in a Koehler corrosion bath.

### 3.6. Catalyst Recovery

After the catalytic tests, the catalyst was recovered by centrifugation. Then, the catalyst was subjected to two recovery routes. Route (1) consisted only of washing with portions of ethyl alcohol and hexane, and then drying at 60 °C for 12 h. In Route (2), the catalyst was subjected to the combination of the washing process used in Route (1) with thermal reactivation in a tubular oven at 400 °C for 1 h in an atmosphere of N_2_. The catalyst recovered by both routes was reused in successive transesterification reactions of soybean oil under optimal conditions.

### 3.7. Catalyst Regeneration

As the catalyst demonstrated a reduction in its catalytic activity during the reuse study, the feasibility of employing the regeneration process using sodium silicate in an amount that would allow the concentration of 10% (*w*/*w*) of sodium in the final composition of the catalyst was evaluated. The regeneration process was performed by wet impregnation according to the procedures described by Alsharif et al. [71] and Bitonto et al. [29], with minor adaptations. Initially, 0.23 g of sodium silicate was dissolved in 20 mL of distilled water and then the catalyst previously used, activated at 105 °C, was slowly added. Then, the suspension was kept in an ultrasonic bath at 25 °C for 2 h, followed by magnetic stirring for 2 h. Then, the mixture was dried in an oven at 105 °C for 12 h, followed by heat treatment in a tubular oven at 400 °C for 1 h under flow of 80 mL min^−1^ of N_2_. The regenerated catalyst was evaluated for its reusability.

## 4. Conclusions

The present study proposed the synthesis of a heterogeneous basic catalyst based on biochar produced from the residual biomass of murici functionalized with sodium for application in the synthesis of biodiesel. The characterizations performed by CHNS, TG/DTG, XRD, FTIR, SEM, EDS and basicity confirmed that the impregnation process used in the synthesis of the catalyst was effective. The synthesized catalyst 15Na/BCAM showed the best results during the study, obtaining a biodiesel with an ester content of 97.20% ± 0.31, as well as in accordance with the ASTM D6751 norm, using optimal reaction conditions: temperature of 75 °C, reaction time of 1.5 h, catalyst concentration of 5% (*w*/*w*) and MeOH:oil molar ratio of 20:1. The catalyst 15Na/BCAM was used in five reaction cycles, maintaining biodiesels with ester contents above 65%. In addition, the catalyst was subjected to the regeneration process resulting in increased catalytic activity, providing five more reaction cycles and obtaining biodiesels with ester contents above 75%. Thus, the heterogeneous catalyst based on biochar functionalized with sodium proved to be suitable for the application in the transesterification reaction of soybean oil via methyl route, and applicable for reuse and regeneration processes, indicating a promising alternative to low-cost waste raw materials for the synthesis of basic heterogeneous catalysts.

## Figures and Tables

**Table 1:** Elemental composition of the murici seed, BCAM support and catalyst 15Na/BCAM.

Sample	C	H	N	O ^a^	Na ^b^	Si ^b^
Murici seed	45.17 ± 0.04	2.88 ± 0.02	2.82 ± 0.01	49.07	-	-
BCAM	71.72 ± 0.03	1.49 ± 0.01	1.98 ± 0.01	24.76	-	-
Catalyst 15Na/BCAM	53.85 ± 0.03	1.33 ± 0.01	0.87 ± 0.01	28.45	9.53	5.82

^a^ Determined by difference (C + O + N + H = 100%) per Lim et al. [44]. ^b^ Not detected, determined by difference and stoichiometric ratio.

**Table 2:** Physicochemical properties of soybean biodiesel synthesized using the catalyst 15Na/BCAM and the limits stipulated by ASTM D6751 standard.

Biodiesel Properties	Unit	Test Methods	ASTM D6751 Limits	Present Study
Kinematic viscosity (at 40 °C)	mm^2^ s^−1^	ASTM D445 [61]	1.9–6.0	4.47
Density (at 20 °C)	g cm^−3^	ASTM D1298 [62]	0.875–0.900	0.880
Acid value	mg KOH g^−1^	ASTM D664 [63]	0.5 max	0.20
Cold filtre plugging point	°C	ASTM D6371 [64]	NS	0.0
Flash point	°C	ASTM D93 [65]	130 min	150
Copper strip corrosion	-	ASTM D130 [66]	3 max	1a

NS = Not specified.

**Table 3:** Basicity analysis of the fresh catalysts and catalysts used for 5° cycle reactional recovered using Route (2).

Sample	Basicity (mmol g^−1^)
Catalyst fresh	3.911 ± 0.031
Catalyst after 5° reactional cycle	1.420 ± 0.011

**Table 4:** Different types of catalysts heterogeneous basic based on carbon and their synthesis parameters for biodiesel production.

Precursor	Catalyst	Synthesis of Catalyst				Reactional Conditions				Ester Content (%)	Cycles	References
		Carbonization		Functionalization		T (°C)	t (h)	Catalyst (wt%)	RM (MeOH:oil)			
		T (°C)	t (h)	T (°C)	t (h)							
Avocado seeds	20 wt%. Ca loaded	900	2	900	2	99.5	5.0	7.3	15.6:1	99.50	3	[29]
Banana peel	30K/BP-600	600	2	600	4	65.0	2.0	4.0	15:1	98.91	5	[32]
Pomelo peel	25K/AP-600	600	2	600	3	65.0	2.5	5.0	8:1	98.00	8	[31]
Date seeds	SrO-carbon	400	5	450	4	65.0	1.5	4.0	15:1	94.27	9	[21]
Sargassum oligocystum	Biochar/CaO/K_2_CO_3_	350	2	500	3	65.0	3.3	4.0	18:1	98.83	9	[49]
Rice husk	30Ca/RB700	700	3	700	4	65.0	3.0	8.0	9:1	94.40	10	[30]
Urea	CaO-MgO-800-5	800	5	800	5	70.0	4.4	8.0	21:1	91.10	4	[69]
Commercial activated carbon	CaO/AC	−	−	450	1	190.0	1.35	5.5	15:1	80.98	3	[70]
Murici seed	15Na/BCAM	600	1	400	1	75	1.5	5.0	20:1	97.20	10	**This study**

## Data Availability

Data are contained within the article and Supplementary Materials.

## Supplementary Materials

The following supporting information can be downloaded at: https://www.mdpi.com/article/10.3390/molecules28247980/s1, Figure S1: EDS chemical composition and elementary mapping of the chemical elements on the surface Catalyst after 5° reactional cycle.
