# Supplementary material for: Functionalized Biochar from the Amazonian Residual Biomass Murici Seed: An Effective and Low-Cost Basic Heterogeneous Catalyst for Biodiesel Synthesis
Source: Molecules. 2023 Dec 7;28(24):7980. doi: 10.3390/molecules28247980 (PMC10746047; doi:10.3390/molecules28247980)
Supplement: Supplementary file 1 [file molecules-28-07980-s001.zip › molecules-2701962-supplementary.pdf]

Functionalized Biochar from the Amazonian Residual Biomass Murici Seed: An Effective and Low-Cost Basic Heterogeneous Catalyst for Biodiesel Synthesis

Thaissa Saraiva Ribeiro, Matheus Arrais Gonçalves, Geraldo Narciso da Rocha Filho and Leyvison Rafael Vieira da Conceição *

Laboratory of Catalysis and Oleochemical, Institute of Exact and Natural Sciences, Federal University of Pará, Belém 66075-110, Pará, Brazil; saraivathaissa@gmail.com (T.S.R.); matheusarrais38@gmail.com (M.A.G.); narciso@ufpa.br (G.N.d.R.F.)
* Correspondence: rafaelvieira@ufpa.br

Supplementary material

Figure S1. EDS chemical composition and elementary mapping of the chemical elements on the surface Catalyst after 5° reactional cycle.

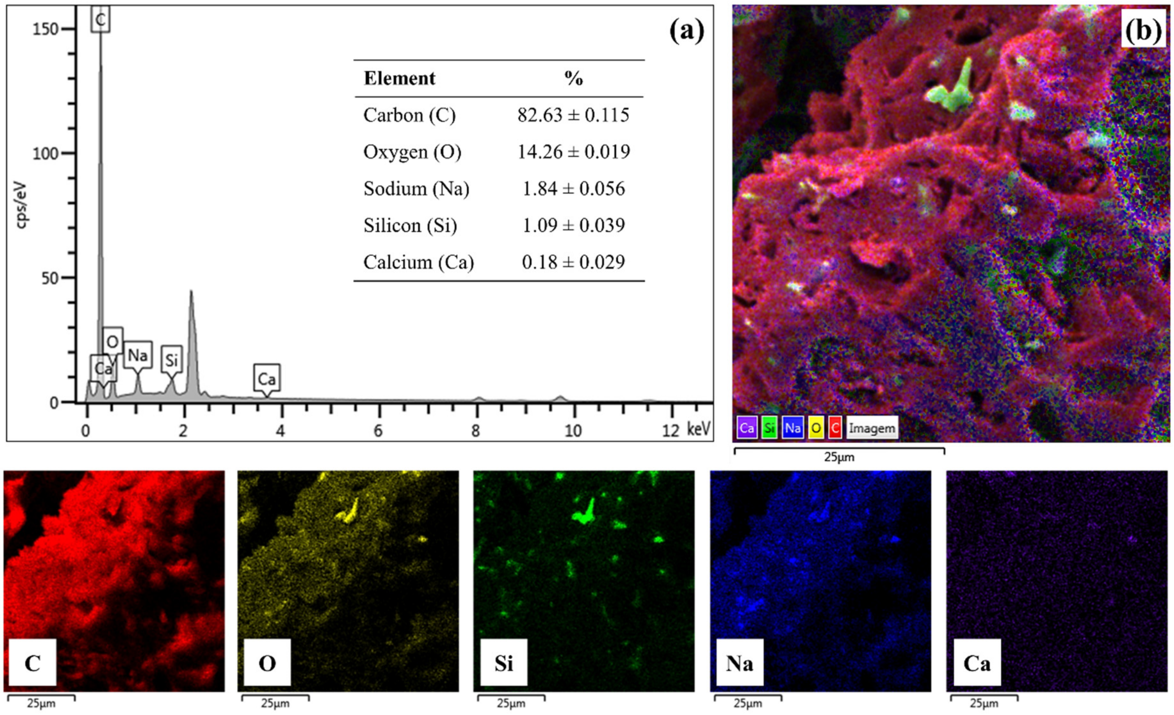

Element	%
Carbon (C)	82.63 ± 0.115
Oxygen (O)	14.26 ± 0.019
Sodium (Na)	1.84 ± 0.056
Silicon (Si)	1.09 ± 0.039
Calcium (Ca)	0.18 ± 0.029
